# Comparative Transcriptome Analysis between Gynoecious and Monoecious Plants Identifies Regulatory Networks Controlling Sex Determination in *Jatropha curcas*

**DOI:** 10.3389/fpls.2016.01953

**Published:** 2017-01-17

**Authors:** Mao-Sheng Chen, Bang-Zhen Pan, Qiantang Fu, Yan-Bin Tao, Jorge Martínez-Herrera, Longjian Niu, Jun Ni, Yuling Dong, Mei-Li Zhao, Zeng-Fu Xu

**Affiliations:** ^1^Key Laboratory of Tropical Plant Resources and Sustainable Use, Xishuangbanna Tropical Botanical Garden, Chinese Academy of SciencesMenglun, China; ^2^Instituto Nacional de Investigaciones Forestales, Agrícolas y PecuariasHuimanguillo, Mexico

**Keywords:** female plant, floral development, inflorescence, physic nut, phytohormone, sex expression

## Abstract

Most germplasms of the biofuel plant *Jatropha curcas* are monoecious. A gynoecious genotype of *J. curcas* was found, whose male flowers are aborted at early stage of inflorescence development. To investigate the regulatory mechanism of transition from monoecious to gynoecious plants, a comparative transcriptome analysis between gynoecious and monoecious inflorescences were performed. A total of 3,749 genes differentially expressed in two developmental stages of inflorescences were identified. Among them, 32 genes were involved in floral development, and 70 in phytohormone biosynthesis and signaling pathways. Six genes homologous to *KNOTTED1-LIKE HOMEOBOX GENE 6* (*KNAT6*), *MYC2*, *SHI-RELATED SEQUENCE 5* (*SRS5*), *SHORT VEGETATIVE PHASE* (*SVP*), *TERMINAL FLOWER 1* (*TFL1*), and *TASSELSEED2* (*TS2*), which control floral development, were considered as candidate regulators that may be involved in sex differentiation in *J. curcas*. Abscisic acid, auxin, gibberellin, and jasmonate biosynthesis were lower, whereas cytokinin biosynthesis was higher in gynoecious than that in monoecious inflorescences. Moreover, the exogenous application of gibberellic acid (GA_3_) promoted perianth development in male flowers and partly prevented pistil development in female flowers to generate neutral flowers in gynoecious inflorescences. The arrest of stamen primordium at early development stage probably causes the abortion of male flowers to generate gynoecious individuals. These results suggest that some floral development genes and phytohormone signaling pathways orchestrate the process of sex determination in *J. curcas*. Our study provides a basic framework for the regulation networks of sex determination in *J. curcas* and will be helpful for elucidating the evolution of the plant reproductive system.

## Introduction

Flowering plants show extreme diversity in their reproductive systems. The determination of the environmental and genetic factors that control sexual diversification is a central problem in plant evolutionary biology (Barrett, 2002). Plant sex determination that leads to the physical separation of male and female reproductive organs plays a crucial role in the evolutionary process from hermaphroditic to unisexual flowers (Dellaporta and Calderon-Urrea, 1993; Tanurdzic and Banks, 2004). In order to facilitate outcrossing and more efficient allocation of resources to optimization of reproduction (Bawa, 1980; Thomson and Barrett, 1981), approximately 10% of angiosperm species have produced unisexual flower reproductive systems in dioecious and monoecious plant taxa (Yampolsky and Yampolsky, 1922). Genetic, hormonal, and environmental cues are involved in the regulation of sex determination in flowering plants (Aryal and Ming, 2014). Sex determination has evolved multiple times independently suggesting that various different genetic mechanisms underlie unisexual flower development (Ainsworth et al., 1998; Chuck, 2010; Diggle et al., 2011).

In cucumber, sex determination is controlled by three genetic loci: *F*, *A*, and *M*. The *F* locus controls the degree of femaleness, the *A* locus increases maleness and the *M* locus is responsible for the selective abortion of stamens (Pierce and Wehner, 1990; Perl-Treves, 1999). The *F* locus is linked to the *1-aminocyclopropane-1 carboxylic acid synthase* (*ACS*) gene encoding the rate-limiting enzyme in ethylene (ETH) biosynthesis, which is present as a single copy in monoecious lines but as a duplicated copy in gynoecious lines (Kamachi et al., 1997; Mibus and Tatlioglu, 2004). The *M* locus encodes the *CsACS2* gene, in which a conserved residue conversion (Gly33Cys) causes bisexual flower production (Li et al., 2009). The ETH receptor *ETR1* is suppressed, causing female flower generation in *Arabidopsis*, suggesting that ETH perception is involved in stamen abortion (Wang et al., 2010). Sex determination in melon is controlled by two loci, *andromonoecious* (*a)* and *gynoecious* (*g)* (Poole and Grimball, 1939), and their interaction results in a range of sexual types (Kenigsbuch and Cohen, 1990). The *a* locus is linked to the *GmACS-7* gene, in which a single missense mutation causes loss of enzymatic activity, resulting in the development of male organs to generate andromonoecious plants (Boualem et al., 2008). CmACS-7 mainly represses stamen development and is not required for carpel development, although CmACS-7 is expressed in the carpel primordia during melon flower development (Boualem et al., 2008). The *g* locus is links to a transcription factor, CmWIP1, which is a repressor of carpel development. The activation of *CmWIP1* by promoter methylation results in the transition from male to female flowers in gynoecious plants (Martin et al., 2009). *CmACS-11*, encoding a key enzyme of ETH biosynthesis, represses *CmWIP1* expression, and *CmACS-11* loss-of-function mutation lead to the transition from monoecious to androecious individuals (Boualem et al., 2015). *CmACS-11, CmWIP1*, and *CmACS-7* coregulate sex determination in melon. In maize, seven mutants affecting male flower (tassel) development have been identified, including six *tasselseed* mutants and one *na1* mutant (Zhang J. et al., 2014). *tasselseed1* (*ts1*) encodes a lipoxygensae involved in jasmonic acid (JA) biosynthesis. Loss of functional *ts1* causes a decrease in endogenous JA concentrations in developing inflorescences, and exogenous JA can rescue stamen development in *ts1* mutants (Acosta et al., 2009). *tasselseed2* (*ts2*) encodes a short-chain alcohol dehydrogenase, required for the arrest of pistil primordia development in the maize tassel (DeLong et al., 1993). *tasselseed4* (*ts4*) encodes a miR172 microRNA targeting *tasselseed6* (*ts6*)/*indeterminate spikelet1* (*ids1*), which is an *APETALA2* (*AP2*)-like gene. *ts4* and *ids1* are required for the carpel abortion in the tassel (Chuck et al., 2007). *nana plant1* (*na1*) encodes a 5α-steroid reductase involved in brassinosteroid (BR) biosynthesis, and a mutation of *na1* causes a decrease in BR metabolites, leading to dwarf and feminizing phenotypes (Hartwig et al., 2011). In addition, *anther ear* (*an1*), *dwarf1* (*d1)*, *d2*, *d3*, *d5*, and *d8* mutants, which are deficient in gibberellic acid (GA) biosynthesis or perception, produce perfect florets in the tassel (Dellaporta and Calderon-Urrea, 1994), and GA application to *an1* converts male to gynoecious florets in the tassel (Bensen et al., 1995), indicating that GA is a negative regulator during stamen development in maize (Tanurdzic and Banks, 2004; Chen and Tan, 2015). However, GA is a positive regulator in the development of stamens and pollen in *Arabidopsis* and rice (Cheng et al., 2004) and is considered to exert masculine effects in plant sex expression (Aryal and Ming, 2014). In *Diospyros*, the autosomal *MeGI* (Japanese for “female tree”) is a feminizing gene controlling anther fertility in a dosage-dependent fashion. A Y-chromosome *OGI* (Japanese for “male tree”) encoding a small RNA suppresses *MeGI* expression, resulting in the generation of androecious individuals (Akagi et al., 2014). These results suggest that at least three different regulation mechanisms of sex determination are present in flowering plants.

The exogenous application of auxin, BR, cytokinin (CK), ETH, GA, JA, and their inhibitors affects sex expression in flowering plants (Louis and Durand, 1978; Chailakhyan, 1979; Durand and Durand, 1984; Irish and Nelson, 1989; Perl-Treves, 1999), and some of them have opposing effects on sex determination in different plant lineages (Yamasaki et al., 2005). Environmental cues such as temperature, photoperiod, nutrition, drought, pH, and seasonality also effect sex determination in many species (Korpelainen, 1998; Field et al., 2013; Golenberg and West, 2013). However, the mechanism of environmental sex determination remains largely unknown, and epigenetic mechanisms are likely involved in the process (Piferrer, 2013).

*Jatropha curcas* L. is known as a potential biofuel plant, owing to its high seed oil content (Fairless, 2007; Sato et al., 2011). Most *J. curcas* germplasms are monoecious (Rincon-Rabanales et al., 2016), bearing male and female flowers on the same inflorescence. Low female:male flower ratio of approximately 1:13–29 in monoecious plants has been considered to be one of the factors causing low seed yield in *J. curcas* (Pan and Xu, 2011). Therefore, it is crucial to improve the female flower number per inflorescence for the increase in *J. curcas* seed yield. Previous research showed that the exogenous application of CK (6-benzyladenine, BA) on inflorescence buds significantly increases the female flower number and female:male flower ratio, resulting in an increase in seed yield (Pan and Xu, 2011; Chen et al., 2014; Fröschle et al., 2016). These results suggest that CKs are involved in *Jatropha* sex differentiation. In this study, a gynoecious genotype of *J. curcas*, whose male flowers are aborted at early stage of inflorescence development, was used for a comparative transcriptome analysis between gynoecious and monoecious inflorescences, to identify the regulation networks that participate in sex differentiation of *J. curcas.* The findings of this study will be helpful in understanding the sex determination mechanism in *J. curcas*.

## Materials and Methods

### Application of Gibberellin (GA) and Collection of Samples for RNA Isolation

**FIGURE 1:**
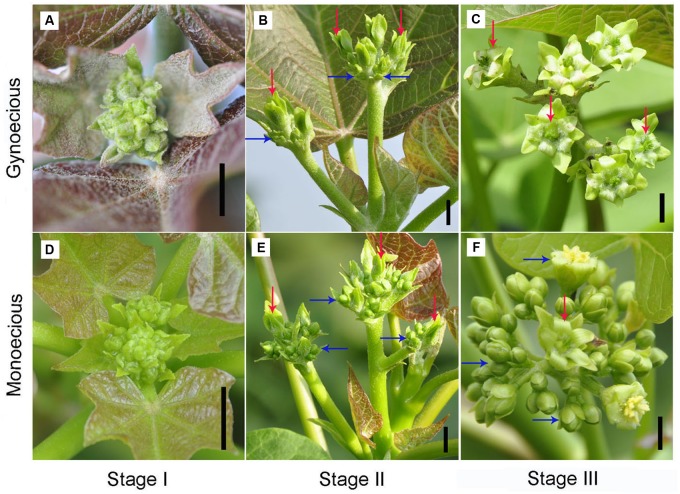
**Morphological comparison of inflorescences between gynoecious and monoecious plants at different developmental stages.** **(A–C)** Indicate gynoecious inflorescences at stages I, II, and III, respectively. **(D–F)** Indicate monoecious inflorescences at stages I, II, and III. Stage I spans approximately 1–7 days from the formation of visible inflorescence buds in which female and male flowers are normally develop in gynoecious plants. Stage II spans approximately 8–20 days from the formation of visible inflorescence buds in which female flowers are normally develop and male flowers are arrest in gynoecious plants. Stage III spans approximately 21–30 days from the formation of visible inflorescence buds in which gynoecious inflorescences have blooming female flowers. Red arrows indicate female flowers and blue arrows indicate male flowers; bar, 0.5 cm.

Gynoecious and monoecious plants were grown in the field in the Xishuangbanna Tropical Botanical Garden of the Chinese Academy of Sciences, located in Menglun town in Mengla County (21° N, 101° E), Yunnan province, China. Gynoecious inflorescence buds that occurred about 1–7 days were treated with 1.5 mM GA_3_ solution. Inflorescence buds that occurred about 3–4 days (stage I) and 8–9 days (stage II) were harvested from gynoecious and monoecious plants, respectively, for RNA isolation (**Figure 1**). Three biological replications were performed for each sample group. Morphologies of female and male flowers at developmental stages I and II were observed by a 3D super depth digital microscope (ZEISS Smartzoom 5).

### RNA Isolation and Transcriptome Sequencing

Total RNA was extracted from inflorescences using the plant total RNA extraction reagent pBIOZOL (Bioflux, Germany). RNA purity was checked with a NanoPhotometer spectrophotometer (IMPLEN, Westlake Village, CA, USA). RNA concentration was determined with a Qubit 2.0 Fluorometer (Life Technologies, Carlsbad, CA, USA). RNA integrity was assessed with an Agilent Bioanalyzer 2100 system (Agilent Technologies, Santa Clara, CA, USA). Sequencing libraries were prepared with a NEBNext Ultra RNA Library Prep Kit for Illumina (NEB, USA) following the manufacturer’s specifications. Library quality was assessed on the Agilent Bioanalyzer 2100 system. The clustering of the index-coded samples was performed on a cBot Cluster Generation System according to the manufacturer’s instructions. After cluster generation, the libraries were sequenced on an Illumina HiSeq 2500 platform and 125-base paired-end reads were produced. Sequencing was performed by Novogene Bioinformatics Technology (Beijing, China).

### *De novo* Transcriptome Assembly and Abundance Estimation

Low-quality (with < 20 Phred scores) reads in raw reads were removed with Fastq_clean (Zhang M. et al., 2014) and assessed with FASTQC^1^. Clean reads were assembled using Trinity (version 2.0.6) with default parameters (Grabherr et al., 2011; Haas et al., 2013). The paired-end reads from each library were mapped to the *de novo* assemblies using Bowtie (version 1.1.1) (Langmead et al., 2009), and abundance estimation of transcripts was performed using Corset (version 1.03) (Davidson and Oshlack, 2014).

### Identification of Differentially Expressed Transcripts

Cluster-level count data generated from Corset were processed with the edgeR package (Robinson et al., 2010). Very low-count transcripts were removed and transcripts with at least one count per million reads (CPM) in at least three libraries were kept for analysis. A design matrix fitting generalized linear models (GLMs) was constructed and the Cox–Reid profile-adjusted likelihood (CR) method was used to estimate common, trended, and tagwise dispersions. Differential expressed transcripts were determined using GLM likelihood ratio test and transcripts with a false discovery rate (FDR) of <0.001 were reported for annotation analysis. A Venn diagram of differentially expressed genes was generated using Venny diagrams^2^.

### Transcript Annotation and Clustering Analysis

Gene Ontology (GO) classification and enrichment analysis were performed with DAVID (Huang et al., 2009). Hierarchical clustering of genes was performed using the pheatmap R package (version 1.0.7)^3^.

### Real Time Quantitative PCR (qPCR)

Total RNA was extracted using the plant total RNA extraction reagent pBIOZOL (Bioflux, Germany). For each sample, total RNA (1 μg) was used for cDNA synthesis using PrimeScript kit (Takara Biotechnology, China). qPCR was performed on Roche Light Cycler 480II system (Roche) using LightCycler FastStart DNA MasterPLUS SYBR Green I Kit (Roche). Each sample has two or three biological replicates. Primers used for qPCR analysis were listed in **Supplementary Table S7**.

### Availability of Supporting Data

All RNA-Seq data for this project have been deposited in NCBI under the SRA accession SRP092157. For simplicity, only sequences of genes that appeared in the context of this paper are listed in **Supplementary Data Sheet 1**.

## Results

### Morphological Comparison between Gynoecious and Monoecious Plants

**FIGURE 2:**
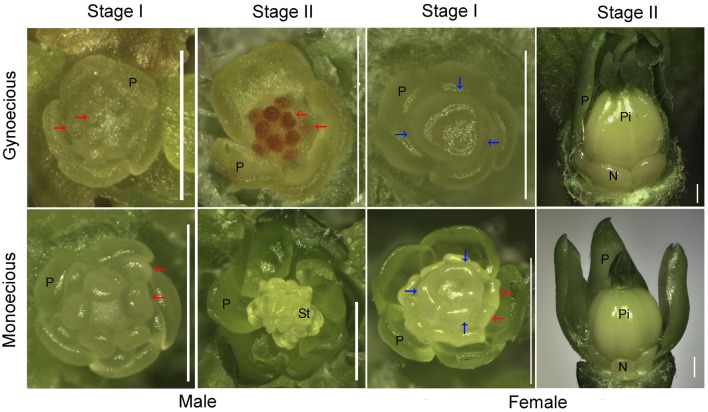
**Morphological comparison of female and male flowers between gynoecious and monoecious plants at developmental stages I and II.** P, petal; St, stamen; Pi, pistil; N, nectary; red and blue arrows indicate stamen and carpel primordia, respectively; bar, 0.5 mm.

To facilitate comparative research on inflorescence development between gynoecious and monoecious plants, the process of inflorescence development was roughly classified into three stages on the basis of inflorescence morphology. In stage I, spanning 1–7 days from the formation of visible inflorescence buds, both female and male flowers could develop in gynoecious plants that is similar to the monoecious plants (**Figure 1**). In this stage, 10 stamen primordia were found in an inner-outer whorl arrangement in male flowers, and three carpel primordia emerged in female flowers in both gynoecious and monoecious plants (**Figure 2**). Interestingly, stamen primordia also presented in monoecious female flowers, but were invisible in gynoecious ones (**Figure 2**). In stage II, spanning 8–20 days, the development of male flowers in gynoecious plants was clearly arrested and stamen primordia turned brown, whereas in monoecious male flowers stamen primordia developed into anthers (**Figures 1** and **2**). In this stage, carpels closed up to develop into ovaries, and nectaries were formed in both gynoecious and monoecious female flowers; stamen primordia in monoecious female flowers were degenerated (**Figure 2**). In stage III, spanning 21–30 days, gynoecious inflorescences had blooming female flowers and vestigial male flowers, whereas monoecious inflorescences consisted of normal female and male flowers (**Figure 1**). Moreover, leaves of gynoecious plants were larger than ones of monoecious plants; gynoecious fruits were spindle-shaped, whereas monoecious fruits are round, but their seeds are similar in size and shape (**Supplementary Figures S1** and **S2**).

### Identification of Differentially Expressed Genes

**FIGURE 3:**
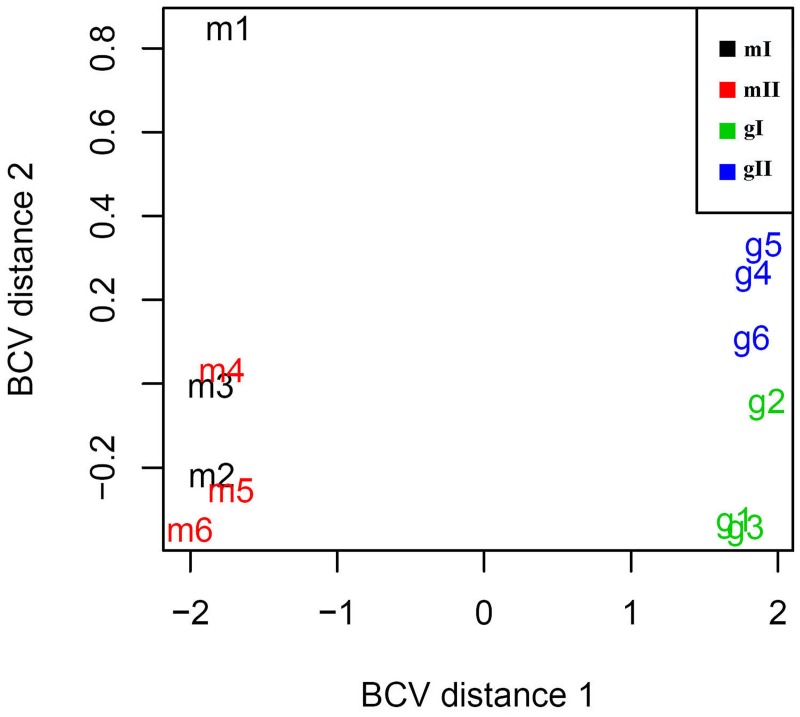
**Relationships between 12 inflorescence samples based on multidimensional scaling.** In the plot, dimension 1 clearly separates monoecious and gynoecious samples. Dimension 2 unambiguously separates two different developmental phase samples in gynoecious plants, whereas two group monoecious samples cannot be clearly separated. mI containing m1, m2, and m3, indicates monoecious samples at stage I; mII containing m4, m5, and m6, indicates monoecious samples at stage II; gI containing g1, g2, and g3, indicates gynoecious samples at stage I; gII containing g4, g5, and g6, indicates gynoecious samples at stage II. BCV, biological coefficient of variation.

**FIGURE 4:**
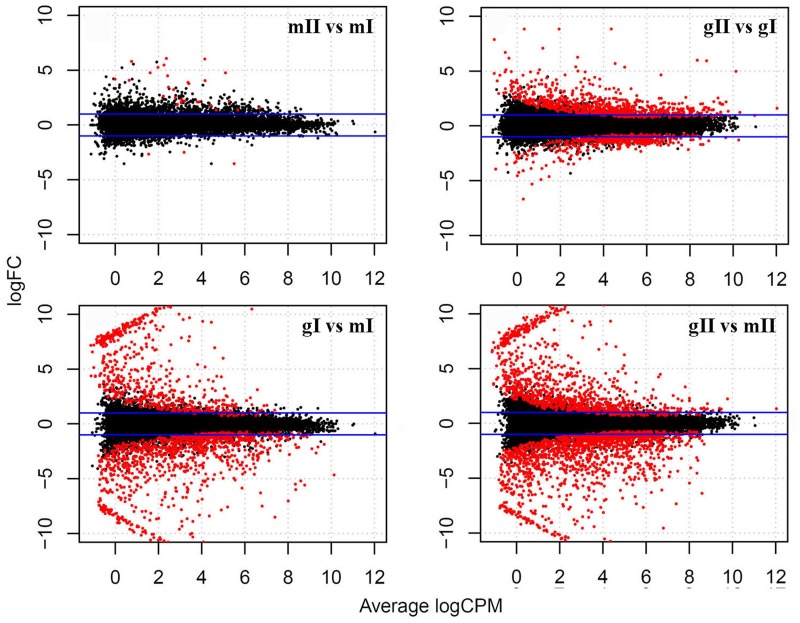
**Differentially expressed genes in the pairwise mII vs. mI, gII vs. gI, gI vs. mI and gII vs. mII in *J. curcas.* mII vs. mI indicates the pairwise stage II vs. I in monoecious inflorescences; gII vs. gI indicates the stage II vs. I in gynoecious inflorescences; gI vs. mI indicates the gynoecious vs. monoecious inflorescences at stage I and gII vs. mII indicates the gynoecious vs. monoecious inflorescences at stage II.** Blue lines indicate genes with a twofold change; red points indicate genes with a significantly different expression at a false discovery rate (FDR) of <0.001. FC, fold change; CPM, counts per million mapped reads.

To investigate the mechanism of sex determination in *J. curcas*, we collected four groups of samples containing monoecious inflorescences at stages I and II (mI and mII) and gynoecious inflorescences at stages I and II (gI and gII) for RNA-Seq analysis to identify differentially expressed genes that might be involved in the transition between monoecy and gynoecy (**Supplementary Table S1**). A plot that shows the relationship between the four groups of samples based on multidimensional scaling with the biological coefficient of variation, displays that gynoecious and monoecious samples are well separated, indicating the presence of large numbers of differentially expressed genes (**Figure 3**). For simplicity, we will hereafter refer to the comparison of stage II vs. stage I in monoecious inflorescences as the pairwise mII vs. mI, the stage II vs. I in gynoecious inflorescences as pairwise gII vs. gI, the gynoecious vs. monoecious inflorescence at stage I as pairwise gI vs. mI, and the gynoecious vs. monoecious inflorescence at stage II as pairwise gII vs. mII. In all, 3,707 genes were significantly changed at an FDR of <0.001 in four pairs (**Supplementary Table S2**). In pairwise mII vs. mI, only three differentially expressed genes were upregulated and 28 were downregulated, whereas 492 were upregulated and 759 were downregulated in pairwise gII vs. gI (**Figure 4**). In pairwise gI vs. mI, 1,037 genes were upregulated and 630 were downregulated, whereas 1,560 were upregulated and 1,072 were downregulated in pairwise gII vs. gI (**Figure 4**).

**FIGURE 5:**
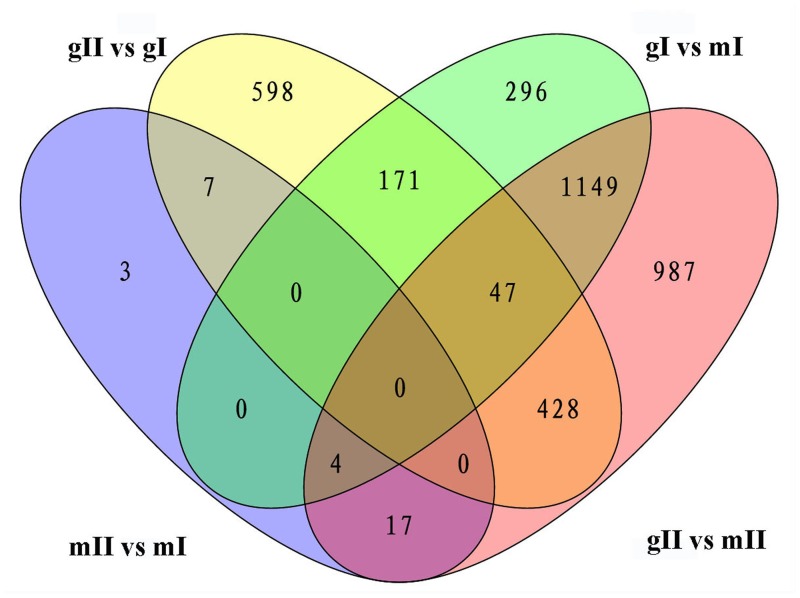
**Venn diagram shows the overlap of differentially expressed genes in the pairwise mII vs. mI, gII vs. gI, gI vs. mI, and gII vs. mII in *J. curcas.*** mII vs. mI, gII vs. gI, gI vs. mI, and gII vs. mII indicate the same pairs as shown in **Figure 4**.

**FIGURE 6:**
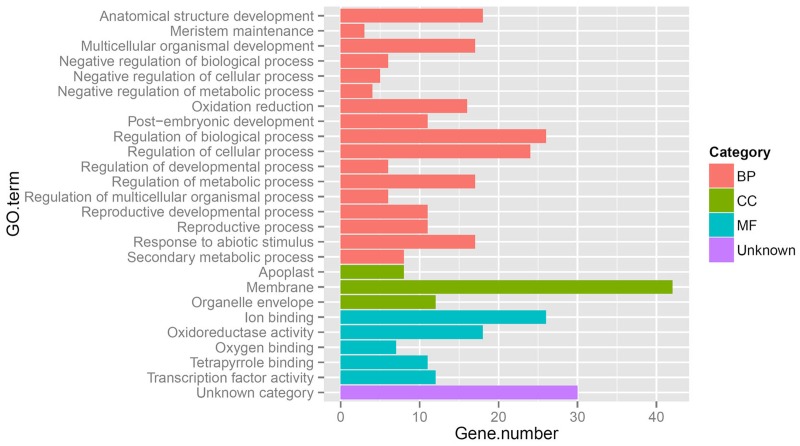
**Gene Ontology (GO) categories of 171 coexpression genes in the pairwise gII vs. gI and gI vs. mI.** BP, biological process; CC, cellular component; MF, molecular function. gII vs. gI and gI vs. mI indicate the same pairs as shown in **Figure 4**.

The above results show that significantly more differentially expressed genes were found during inflorescence development from stage I to II in gynoecious plants than ones in monoecious plants, indicating that a very different physiological and biochemical process was occurring during this developmental transition between these two genotypes. Considering that the morphological differences between gynoecious and monoecious plants were mainly resulted from the arrest of stamen primordia that initiates at stage I (**Figure 2**) and the abortion of male flowers that occurs from stage I to stage II in gynoecious plants (**Figure 1**), we focused on the 171 coexpressed genes in pairwise gI vs. mI and gII vs. gI, excluding the 47 coexpressed genes in pairwise gII vs. gI, gI vs. mI and gII vs. mII (**Figure 5**) because these differentially expressed genes in pairwise gII vs. mII might be a consequence rather than a cause of stamen primordia arrest. GO analysis shows that the 171 coexpressed genes belong to 25 subset GO categories, and the “Membrane,” “Regulation of biological process,” “Regulation of cellular process,” and “Ion binding” functional categories are predominant (**Figure 6**; **Supplementary Table S3**). The genes associated with reproductive (GO:0022414), reproductive developmental (GO:0003006), and developmental regulation (GO:0050793) processes are likely to play important roles during male flower abortion in gynoecious *Jatropha*.

### Differentially Expressed Genes Related to Floral Development

**FIGURE 7:**
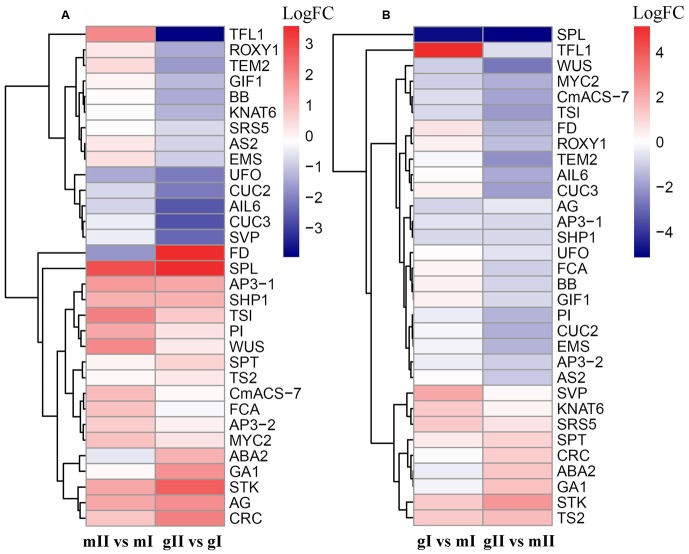
**Hierarchical clustering of differentially expressed genes involved in floral development in the pairwise mII vs. mI, gII vs. gI, gI vs. mI, and gII vs. mII in *J. curcas.*** **(A,B)** Clustering of differentially expressed genes in the pairwise mII vs. mI and gII vs. gI, and in gI vs. mI and gII vs. mII, respectively. mII vs. mI, gII vs. gI, gI vs. mI, and gII vs. mII indicate the same pairs as shown in **Figure 4**.

Owing to that differentially expressed genes associated with the “Reproductive process” and “Reproductive developmental process” functional categories are enriched (*P* < 0.05), we accordingly scanned our assembled data set and identified 32 genes that are homologous to floral development genes in model plants such as *Arabidopsis*, maize, and melon (**Supplementary Table S4**). In pairwise mII vs. mI, 11 genes were upregulated and 2 were downregulated, whereas 8 were upregulated and 11 were downregulated in pairwise gII vs. gI (Fold change ≥ 2.0 and FDR < 0.05) (**Figure 7**; **Supplementary Table S5**). *AGAMOUS* (*AG*), *APETALA3* (*AP3*), *SHATTERPROOF 1* (*SHP1*), *SPOROCYTELESS* (*SPL*), *SEEDSTICK* (*STK*), *FLOWERING LOCUS D* (*FD*), and *UNUSUAL FLORAL ORGANS* (*UFO*) have similar expression patterns in pairwise mII vs. mI and gII vs. gI, suggesting that the changes in transcriptional levels may be caused by different development stages. In pairwise gI vs. mI, six genes were upregulated and three were downregulated, whereas six were upregulated and 16 were downregulated in pairwise gII vs. mII (**Figure 7**; **Supplementary Table S5**).

### Differentially Expressed Genes Involved in Phytohormone Biosynthesis and Signaling Pathways

**FIGURE 8:**
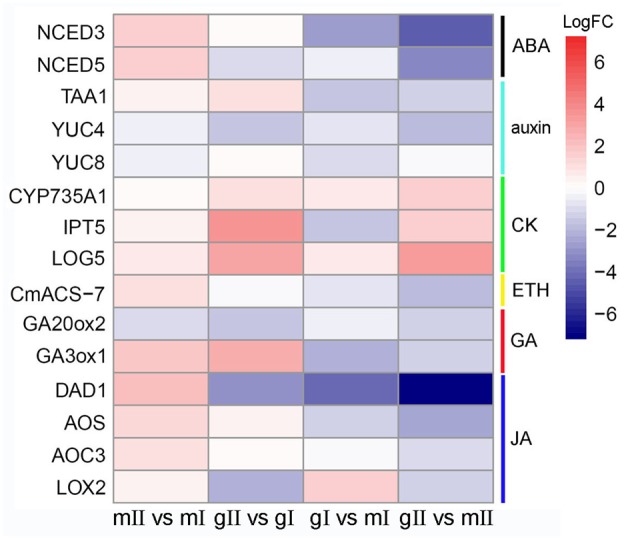
**Expression patterns of differentially expressed genes that encode key enzymes in hormone biosynthesis pathways in the pairwise mII vs. mI, gII vs. gI, gI vs. mI, and gII vs.** mII in *J. curcas.* mII vs. mI, gII vs. gI, gI vs. mI, and gII vs. mII indicate the same pairs as shown in **Figure 4**.

Phytohormones play an important part in the process of sex expression (Aryal and Ming, 2014), we monitored homologous genes involved in hormone metabolism and signaling pathways during the inflorescence development of *J. curcas*. In all, 70 differentially expressed genes were identified, in which eight were from abscisic acid (ABA), 16 from auxin, 9 from BR, 11 from CK, 4 from ETH, 7 from GA, 8 from JA, and 7 from salicylic acid (SA) pathways (**Supplementary Table S6**). Among them, some genes encoding key rate-limiting enzymes in hormone biosynthesis pathways were significantly changed. In pairwise gII vs. gI and gII vs. mII, the expression of NINE-*CIS*-EPOXYCAROTENOID DIOXYGENASES 3 (NCED) and *NCED5* in ABA biosynthesis, TRYPTOPHAN AMINOTRANSFERASE OF ARABIDOPSIS 1 (TAA1) and YUCCA (YUC) in auxin, *1-AMINOCYCLOPROPANE-1-CARBOXYLIC ACID SYNTHASE* (*CmACS-7*) in ETH, *GA 20-oxidase 2* (*GA20ox2*) and *GA 3-oxidase 1* (*GA3ox1*) in GA, *DEFECTIVE ANTHER DEHISCENCE 1* (*DAD1*), *FATTY ACID DESATURASE 8* (*FAD8*), *LIPOXYGENASE 2* (*LOX2*), *ALLENE OXIDE SYNTHASE* (*AOS*), and *ALLENE OXIDE CYCLASE 3* (*AOC3*) in JA were downregulated, whereas *ATP/ADP isopentenyltransferases 5* (*IPT5*), *CYP735A1*, and *LONELY GUY 5* (*LOG5*) in CK were upregulated (**Figure 8**).

### Validation of the Expression Profiles of Candidate Genes Involved in Sex Determination of *J. curcas* by Real Time qPCR

**FIGURE 9:**
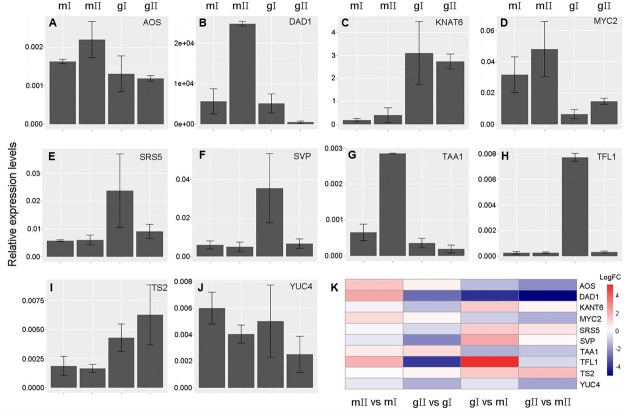
**Validation of expression profiles of the candidate genes involved in sex determination of *J. curcas* by real time qPCR.** **(A)** *ALLENE OXIDE SYNTHASE* (*AOS*). **(B)** *DEFECTIVE ANTHER DEHISCENCE 1* (*DAD1*). **(C)** *KNOTTED1-LIKE HOMEOBOX GENE 6* (*KNAT6*). **(D)** *MYC2*. **(E)** *SHI-RELATED SEQUENCE 5* (*SRS5*). **(F)** *SHORT VEGETATIVE PHASE* (*SVP*). **(G)** *TRYPTOPHAN AMINOTRANSFERASE OF ARABIDOPSIS 1* (*TAA1*). **(H)** *TERMINAL FLOWER 1* (*TFL1*). **(I)** *TASSELSEED2* (*TS2*). **(J)** *YUCCA 4* (*YUC4*). **(K)** Expression patterns of the candidate genes in four pairs. *GAPDH* was used as the internal reference. The mean was calculated from three biological replicates. Error bars show the SD (*n* = 3). mI, mII, gI, and gII indicate the same samples as shown in **Figure 3**. mII vs. mI, gII vs. gI, gI vs. mI, and gII vs. mII indicate the same pairs as in **Figure 4**. FC, fold change.

To validate the expression profiles of candidate genes obtained from the above comparative transcriptome analysis, we selected 10 candidate genes involved in sex determination of *J. curcas* to perform real time qPCR analysis. These 10 genes are *AOS*, *DAD1*, *KNOTTED1-LIKE HOMEOBOX GENE 6* (*KNAT6*), *MYC2*, *SHI-RELATED SEQUENCE 5* (*SRS5*), *SHORT VEGETATIVE PHASE* (*SVP*), *TAA1*, *TERMINAL FLOWER 1* (*TFL1*), *TASSELSEED2* (*TS2*) and *YUCCA 4* (*YUC4*). The results revealed by real time qPCR (**Figure 9**) show that the expression patterns of these genes were consistent with the ones obtained by transcriptome analysis (**Figure 7**; **Supplementary Tables S4** and **S5**), indicating transcriptome data in this study are reliable.

### Effect of GA on Flower Organ Development in Gynoecious Plants

**FIGURE 10:**
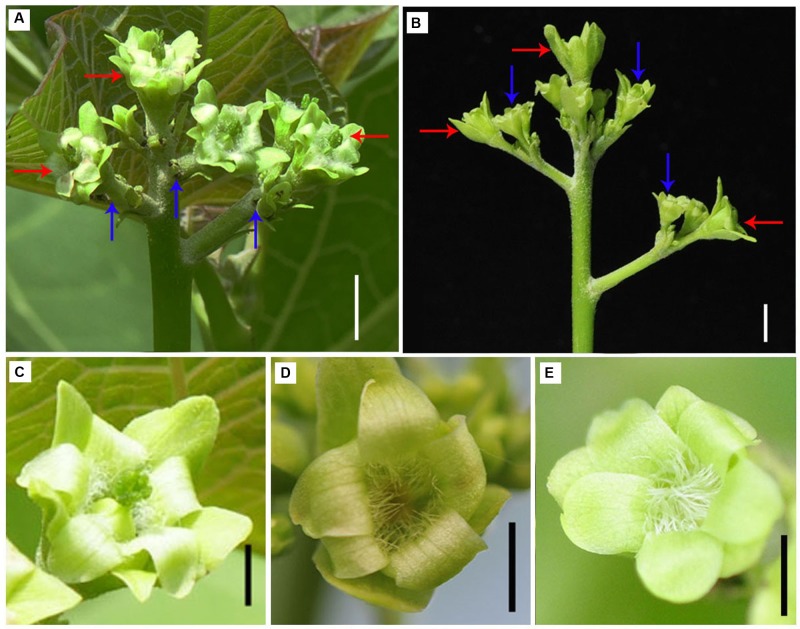
**Application of GA_3_ promotes perianth development in male flowers and prevents pistil development in female flowers in gynoecious inflorescences. (A)** Gynoecious inflorescence without treatment. Red arrows indicate female flowers and blue arrows indicate aborted male flowers. **(B)** Gynoecious inflorescence with GA_3_ treatment. Red arrows indicate neutral flowers originated from female floral meristem, and blue arrows indicate neutral flowers originated from male floral meristem. **(C)** Female flower as shown and indicated with red arrows in **(A)**. **(D)** Neutral flower originated from floral meristem generating female flowers after GA_3_ treatment as shown and indicated with red arrows in **(B)**. **(E)** Neutral flower originated from floral meristem generating male flowers after GA_3_ treatment as shown and indicated with blue arrows in **(B)**. Bar, 1.0 cm.

Since the expression of two GA biosynthesis genes *GA20ox2* and *GA3ox1* was found downregulated in gynoecious inflorescence (**Figure 8**; **Supplementary Table S6**), we investigated the effect of GA on flower organ development in gynoecious plants. The result showed that exogenous application of GA_3_ promoted perianth development in male flowers and partly prevented pistil development in female flowers in gynoecious plants, which resulted in neutral flowers without pistils and stamens in gynoecious inflorescences (**Figure 10**).

## Discussion

### Floral Development Genes May Be Key Regulators in the Transition from Monoecious to Gynoecious Plants

At initiation of inflorescence buds, female and male flower primordia are emerged in both gynoecious and monoecious plants. However, in gynoecious plants, the development of male flowers is aborted, and only female flowers can normally grow; whereas the development of both female and male flowers is normal in monoecious plants. Transcriptome analysis showed that differentially expressed genes in pairwise stage II vs. I in monoecious inflorescence are far less than ones in gynoecious (**Figure 4**). This finding indicates that large physiological and biochemical changes occur in gynoecious inflorescences from stages I to II, in accord with the morphological change of male flower abortion. A large number of differentially expressed genes in pairwise gI vs. mI and gII vs. mII suggest that reproductive system differences between gynoecious and monoecious plants indeed result from inherent genetic variation.

In gynoecious plants, the abortion of male flowers was observed at stage II; however, the initiation of the process is expected to occur at stage I. Thus, the transition from stage I to stage II is a key period determining the sex expression of *J. curcas*. Genes differentially expressed at stage I contribute to the arrest of male flowers in gynoecious inflorescences. As shown in **Supplementary Table S5**, during the development of monoecious inflorescence from stage I to stage II, 11 of the 13 genes involved in the floral development were upregulated at transcriptional level. In contrast, the expression levels of 10 genes involved in the floral development were down-regulated during the development of gynoecious inflorescence from stage I to stage II, whereas the expression of these genes were not significantly changed in monoecious plants (**Supplementary Table S5**). These results suggest that different regulatory mechanisms of inflorescence development are present in monoecious and gynoecious *Jatropha*. The differentially expressed genes with similar expression patterns in pairwise gII vs. gI and gII vs. mII should be caused by male flower abortion, whereas ones with different expression patterns in pairwise gI vs. mI and gII vs. mII, are more likely to contribute to the arrest of stamen primordium in gynoecious plants (**Figure 7**). Among the differentially expressed genes shown in **Figure 7**, *KNAT6*, *MYC2*, *SRS5*, *SVP*, *TFL1*, and *TS2* were considered as strong candidate regulators of sex determination in *J. curcas*.

KNAT6 contributes to the maintenance of the shoot apical meristem and organ separation (Belles-Boix et al., 2006), and is an important regulator of *Arabidopsis* inflorescence architecture (Ragni et al., 2008). MYC2 is a master regulator modulating various JA-dependent signaling in plant development and defense, and controlling the crosstalk between JA and the other hormone signaling pathways (Kazan and Manners, 2013). SRS5 is a member of SHORT-INTERNODES/STYLISH (SHI/STY) family that may be involved in stamen and leaf development and flowering regulation (Staldal et al., 2012). SVP and TFL1 are inhibitors of the transition from vegetative growth to flowering (Hartmann et al., 2000). SVP also represses the biosynthesis of GA at the shoot apex in *Arabidopsis* (Andres et al., 2014). TS2 is a short-chain alcohol dehydrogenase that represses pistil primordium in maize tassels and both *ts1* and *ts2* mutants result in conversion from staminate to pistillate tassels (DeLong et al., 1993). Application of JA to developing tassels can rescue stamen development in *ts1* and *ts2* mutants (Acosta et al., 2009). Our results suggest that some floral development genes and phytohormones (mainly GA and JA) likely coordinate to modulate sex expression in *J. curcas*. It is worth mentioning that the expression levels of homologs of several well-known sex determination genes, such as *CsACS* in cucumber (Li et al., 2009), and *CmACS* and *CmWIP1* in melon (Boualem et al., 2015), were not significantly different between monoecious and gynoecious plants, indicating that the mechanism of sex determination in cucumber and melon is different from that in *J. curcas*.

### GA Plays Important Roles in the Development of *J. curcas* Flowers

GA20oxs and GA3oxs are key enzymes in GA biosynthesis, both being responsible for the conversion from GA_12_ to bioactive GAs (Yamaguchi, 2008). Expression of *GA20ox2* and *GA3ox1* are downregulated in gynoecious inflorescences (**Figure 8**; **Supplementary Table S6**), indicating that the GA biosynthesis is lower in gynoecious than in monoecious inflorescences. Furthermore GA_3_ treatment promoted perianth development in male flowers and repressed pistil development in female flowers to produce neutral flowers in gynoecious inflorescences (**Figure 10**). Our previous studies showed that GA_3_ treatment induced the emergence of neutral and bisexual flowers in monoecious plants (Pi et al., 2013). These results suggest that GA plays essential roles in floral organ development in *J. curcas*. GA treatment did not resume the development of stamens in gynoecious male and female flowers, whereas GA promoted the development of stamens in monoecious female flowers to produce bisexual flowers, implying that the mechanisms controlling the arrest of stamen primordium between gynoecious and monoecious inflorescences are different. In maize, GA is a negative regulator in the development of stamen (Tanurdzic and Banks, 2004; Chen and Tan, 2015), but a positive regulator in *Arabidopsis* and rice (Cheng et al., 2004). Our results showed that GA has multiple functions in floral organ development in *J. curcas*, and also suggested that regulatory mechanisms controlling sex expression are different in various plant species.

### The Biosynthesis of Auxin Is Repressed in Gynoecious Inflorescences

The TAA/YUC two-step pathway is the main auxin biosynthesis pathway (Zhao, 2013) and is widely present in plants (Yunde, 2010). TRYPTOPHAN AMINOTRANSFERASE OF ARABIDOPSIS 1 (TAA1) catalyzes the conversion from L-tryptophan (Trp) to indole-3-pyruvic acid (IPA) (Stepanova et al., 2008), and YUCCA (YUC) flavin-containing monooxygenases (FMOs) catalyze the oxidative decarboxylation of IPA to generate indole-3-acetic acid (IAA) (Mashiguchi et al., 2011). Both TAA1 and FMOs are rate-limiting enzymes in the regulation of auxin biosynthesis. The expression of *TAA1*, *YUC4*, and *YUC8* was downregulated in pairwise gI vs. mI and gII vs. mII, indicating that auxin biosynthesis is lower in gynoecious than in monoecious inflorescences. This finding is supported by the observation that expressions of auxin responsive genes *BTB AND TAZ DOMAIN PROTEIN 2* (*BT2*), *INDOLE-3-ACETIC ACID INDUCIBLE 14* (*IAA14*), *IAA29*, and *SMALL AUXIN UP RNA 20* (*SUR20*), auxin transport genes *PLEIOTROPIC DRUG RESISTANCE 9* (*PPDR9*), and *PIN6* were down-regulated (**Figure 8**; **Supplementary Table S6**). In *Arabidopsis* and barley, high-temperature injury causes a specific decrease of endogenous auxin content in developing anthers, resulting in the arrest of pollen development, and exogenous application of IAA can completely reverse male sterility (Sakata et al., 2011), indicating that auxin is an important regulator in the development of male flowers. This result is in agreement with the finding that auxin biosynthesis is suppressed in gynoecious inflorescences (**Figure 8**), which might result in male flower abortion in gynoecious *Jatropha*.

In addition, auxin plays an indispensable role in regulating inflorescence branching in *Arabidopsis* (Teo et al., 2014). A loss-of-function mutation in PIN-FORMED 1 (PIN1), an auxin carrier, abolishes the formation of an inflorescence meristem, resulting in naked inflorescence stems (Vernoux et al., 2000). In *J. curcas*, gynoecious inflorescences contain fewer branches than monoecious inflorescences (**Figures 1C,F**), which may be associated with the decrease of auxin biosynthesis in gynoecious inflorescence. This suggests that auxin may play a similar role in controlling inflorescence branching in *J. curcas*.

Two *Arabidopsis* cytochrome P450 monooxygenases, CYP79B2 and CYP79B3, catalyze the conversion of Trp to indole-3-acetaldoxime (IAOx), which is a common intermediate for the biosynthesis of IAA, 3-indolylmethyl-glucosinolate (IG) and camalexin (Hull et al., 2000; Mikkelsen et al., 2000; Zhao et al., 2002; Glawischnig et al., 2004). Overexpression of *CYP79B2* increases IAA level leading to auxin overproduction phenotypes of transgenic plants (Zhao et al., 2001, 2002). Correspondingly, *cyp79B2 cyp79B3* double mutants display IAA-deficient phenotypes in *Arabidopsis* (Zhao et al., 2002). Thus, IAOx is an important intermediate in auxin biosynthesis distinct from the TAA/YUC pathway (Zhao et al., 2002; Sugawara et al., 2009). However, IAOx has been detected only in *Arabidopsis* and related species, and the CYP79B family is also limited to Brassicaceae species, suggesting that the IAOx pathway is a Brassicaceae species-specific pathway in IAA biosynthesis (Mashiguchi et al., 2011). In *J. curcas*, the homologs *CYP79B2* and *CYP79B3* are upregulated in pairwise gI vs. mI, indicating that IOAx biosynthesis is higher in gynoecious than in monoecious inflorescences (**Supplementary Table S6**); however, the expression of *NITRILASES 1* (*NIT1*), *NIT2*, and *NIT3*, which are involved in the conversion from IAN to IAA, was not significantly changed (**Supplementary Table S6**). Moreover, the homolog *CYP83B1*, which converts IAOx to aci-nitro compounds in IG biosynthesis (Barlier et al., 2000; Bak et al., 2001), is upregulated in pairwise gI vs. mI and gII vs. mII (**Supplementary Table S6**). These results suggest that IAOx is more likely to be channeled into IG biosynthesis than into IAA, or even that IAOx is not involved in IAA biosynthesis, although an IAOx biosynthesis pathway is present in *J. curcas*.

### JA Signaling Pathway May Participate in the Abortion of Male Flowers

Previous study showed that JA plays an essential role in regulating the development of inflorescences, flowers, and male organs, and JA biosynthesis and signaling pathway during reproductive development are conserved in *Arabidopsis*, tomato, and rice (Yuan and Zhang, 2015). In *Arabidopsis*, *DEFECTIVE ANTHER DEHISCENCE 1* (*DAD1*) encodes a chloroplastic phospholipase A1 that catalyzes the initial step of JA biosynthesis, and the *dad1* mutant displays defects in anther dehiscence, pollen maturation, and flower opening (Ishiguro et al., 2001). *FATTY ACID DESATURASE 8* (*FAD8*), *LIPOXYGENASE 2* (*LOX2*), *ALLENE OXIDE SYNTHASE* (*AOS*), and *ALLENE OXIDE CYCLASE 3* (*AOC3*) are involved in JA biosynthesis in plastids (Turner et al., 2002). In *J. curcas*, the homologs of these genes are all downregulated in pairwise gI vs. mI or/and gII vs. mII, implying that JA biosynthesis is lower in gynoecious than in monoecious inflorescences (**Figure 8**; **Supplementary Table S6**). The expression of *FAD8*, *DAD1*, *AOS*, and *AOC3* is upregulated during inflorescence development from stage I to II in monoecious plants, whereas *DAD1* and *LOX2* are downregulated in gynoecious plants (**Figures 8** and **9**; **Supplementary Table S6**). These results indicate that JA biosynthesis is also conserved in *Jatropha*, suggesting that the decrease of JA biosynthesis is likely to contribute to the abortion of male flowers in gynoecious plants. However, treatment with low concentration (6 μM) of MeJA did not resume the development of stamens of male and female flowers in gynoecious plants (data not shown). Because *Jatropha* is extremely sensitive to MeJA, and treatment with 10 μM of MeJA caused severe injury to inflorescences (data not shown), the concentration of MeJA used for treatment of *Jatropha* inflorescences will be optimized in future studies.

### Regulatory Networks Participate in the Transition from Monoecy to Gynoecy in *J. curcas*

The phytohormones auxin, BR, CK, ETH, GA, and JA influence sex differentiation in flowering plants (Louis and Durand, 1978; Chailakhyan, 1979; Durand and Durand, 1984; Irish and Nelson, 1989; Perl-Treves, 1999). Our results showed that the expression of some key genes involved in ABA, auxin, CK, ETH, GA, and JA biosynthesis pathways was significantly changed during inflorescence development and sex expression in *J. curcas*, suggesting that these hormones may participate in these processes. Thus, it is interesting to investigate how these hormones interact with one another to regulate the abortion of male flowers in gynoecious plants. However, only *MYC2*, a hub gene, was identified. MYC2 is a “master switch” in the crosstalk between JA and the other hormone signaling pathways, and has a wide range of functions in the regulation of developmental programs (Kazan and Manners, 2013). Both GA and JA promote *MYC2* expression by a similar mechanism to modulate downstream gene expression (Kazan and Manners, 2013). Moreover, many reverse genetics experiments support that GA and JA play crucial roles in the development of stamens and pollen (Cheng et al., 2004; Yuan and Zhang, 2015). Based on the results, we speculate that the arrest of stamen primordium, which may result from one or more gene mutation in monoecious *Jatropha*, causes the abortion of male flowers to generate gynoecious individuals, in which some floral development genes and plant hormone signaling pathways participate in.

## Author Contributions

Z-FX, M-SC, and B-ZP designed experiments. M-SC and Z-FX wrote the manuscript. M-SC, LN, and YD analyzed and interpreted sequencing data. QF, Y-BT, JM-H, JN, and M-LZ carried out experiments and reviewed the manuscript.

## Conflict of Interest Statement

The authors declare that the research was conducted in the absence of any commercial or financial relationships that could be construed as a potential conflict of interest.

## Footnotes

1 http://www.bioinformatics.babraham.ac.uk/projects/fastqc

2 http://bioinfogp.cnb.csic.es/tools/venny/index.html

3 https://github.com/cran/pheatmap
