# Supplementary material for: Comparative Transcriptome Analysis between Gynoecious and Monoecious Plants Identifies Regulatory Networks Controlling Sex Determination in Jatropha curcas
Source: Front Plant Sci. 2017 Jan 17;7:1953. doi: 10.3389/fpls.2016.01953 (PMC5239818; doi:10.3389/fpls.2016.01953)
Supplement: Supplementary file 1 [file Table_1.doc]

**Supplementary Table S1** Sequencing read counts, quality, and alignment statistics for 12 *Jatropha* inflorescence samples.

| **ID** | **Clean Pairs** | **Error (%)** | **Q30 (%)** | **Mapped (%)** | **Sample name** |
| --- | --- | --- | --- | --- | --- |
| m1_1 | 24532948 | 0.03 | 92.04 | 88.87 | Monoecious inflorescence  at stage I (mI) |
| m1_2 | 24532948 | 0.04 | 89.41 |
| m2_1 | 24696197 | 0.03 | 92.43 | 88.06 |
| m2_2 | 24696197 | 0.04 | 89.89 |
| m3_1 | 24834405 | 0.03 | 92.23 | 87.94 |
| m3_2 | 24834405 | 0.04 | 89.17 |
| m4_1 | 19628309 | 0.03 | 92.36 | 88.35 | Monoecious inflorescence  at stage II (mII) |
| m4_2 | 19628309 | 0.04 | 89.42 |
| m5_1 | 27121140 | 0.03 | 92.42 | 88.28 |
| m5_2 | 27121140 | 0.04 | 89.06 |
| m6_1 | 21394277 | 0.03 | 92.25 | 87.79 |
| m6_2 | 21394277 | 0.04 | 89.17 |
| g1_1 | 25925411 | 0.03 | 93.17 | 85.70 | Gynoecious inflorescence  at stage I (gI) |
| g1_2 | 25925411 | 0.04 | 89.17 |
| g2_1 | 24357828 | 0.03 | 92.78 | 88.43 |
| g2_2 | 24357828 | 0.03 | 90.97 |
| g3_1 | 31025373 | 0.03 | 93.15 | 85.06 |
| g3_2 | 31025373 | 0.04 | 89.65 |
| g4_1 | 26501895 | 0.03 | 92.63 | 87.63 | Gynoecious inflorescence  at stage II (gII) |
| g4_2 | 26501895 | 0.04 | 89.86 |
| g5_1 | 26792993 | 0.03 | 90.81 | 88.43 |
| g5_2 | 26792993 | 0.03 | 90.82 |
| g6_1 | 25626351 | 0.03 | 91.70 | 85.76 |
| g6_2 | 25626351 | 0.03 | 91.26 |

Mapped read indicates the paired reads with at least one reported alignment.
