# Supplementary material for: Comparative Transcriptome Analysis between Gynoecious and Monoecious Plants Identifies Regulatory Networks Controlling Sex Determination in Jatropha curcas
Source: Front Plant Sci. 2017 Jan 17;7:1953. doi: 10.3389/fpls.2016.01953 (PMC5239818; doi:10.3389/fpls.2016.01953)
Supplement: Supplementary file 5 [file Table_5.doc]

**Supplementary Table S5** Differentially expressed genes involved in the floral development in the pairwise mII vs. mI, gII vs. gI, gI vs. mI, and gII vs. mII in *J. curcas*.

| mII vs mI | gII vs gI | gI vs mI | gII vs mII |
| --- | --- | --- | --- |
| - | ABA2 | - | ABA2 |
| AG | AG | - | - |
| - | AIL6 | - | AIL6 |
| AP3-1 | AP3-1 | - | - |
| - | - | - | AP3-2 |
| - | - | - | AS2 |
| - | BB | - | - |
| CmACS-7 | - | - | CmACS-7 |
| - | CRC | - | CRC |
| - | CUC2 | - | CUC2 |
| - | CUC3 | - | CUC3 |
| - | EMS1 | - | EMS1 |
| FCA | - | - | FCA |
| FD | FD | - | FD |
| - | GA1 | - | GA1 |
| - | GIF1 | - | - |
| - | KNAT6 | KNAT6 | - |
| - | - | MYC2 | MYC2 |
| PI | - | - | PI |
| - | ROXY1 | - | ROXY1 |
| SHP1 | SHP1 | - | - |
| SPL | SPL | SPL | SPL |
| - | - | - | SPT |
| - | - | SRS5 | - |
| STK | STK | STK | STK |
| - | SVP | SVP | - |
| - | TEM2 | - | TEM2 |
| TFL1 | TFL1 | TFL1 | - |
| TS1 | - | - | TS1 |
| - | - | TS2 | TS2 |
| UFO | UFO | - | - |
| WUS | - | WUS | WUS |

mII vs. mI represents the pairwise stage II vs. I in monoecious inflorescences; gII vs. gI represents stage II vs. I in gynoecious inflorescences; gI vs. mI represents gynoecious vs. monoecious inflorescences at stage I, and gII vs. mII represents gynoecious vs. monoecious inflorescences at stage II. Red indicates up-regulated gene; blue indicates down-regulated gene; minus (-) indicates a gene whose expression was not significantly changed.
