# Supplementary material for: Comparative Transcriptome Analysis between Gynoecious and Monoecious Plants Identifies Regulatory Networks Controlling Sex Determination in Jatropha curcas
Source: Front Plant Sci. 2017 Jan 17;7:1953. doi: 10.3389/fpls.2016.01953 (PMC5239818; doi:10.3389/fpls.2016.01953)
Supplement: Supplementary file 7 [file Table_7.doc]

**Supplementary Table S7** List of primers used for qPCR analysis in this study.

| Gene name | Forward primer (5' to 3') | Reverse primer (5' to 3') |
| --- | --- | --- |
| *AOS* | CggTTCTTCTCATggACTggACTCTT | TgCTggTTATgTgggAATAgggTCAA |
| *DAD1* | AATTgggAgggCTTgCTTgACC | CggAgCgAgACAggAgTgAgT |
| *KANT6* | CgACggCggACTACTCAgACA | CTTCTCgTggAATTTCgggAgCAATC |
| *MYC2* | ACggTTCggTgCTAggTTgg | TCTTACggTgCTCCTgCTCTACA |
| *SRS5* | gCCgAgggTTTCAgTgCCAAA | TTgCTgCTggTgTTgCTgTTCTT |
| *TAA1* | gCTgCTCCTTACTACTCggCgTATA | ACCTTTCCCTCTCCTCTgTTCACAA |
| *TFL1* | AAgATCACAAATACAgCACCCACCTT | AACAgCAgCAACAggAAgACCAA |
| *TS2* | AAgACTTgAAggAAAggTTgCTCTgA | CgCTACTTACATCgCAgTggACAT |
| *YUC4* | gCAgAgCCAgTgATACCAgAgATTC | ACTTCCATgCCAgAATTgCCACAT |
| *GAPDH* | TgAAggACTggAgAggTggAAgAgC | ATCAACAgTTggAACACggAAAgCC |
