# Supplementary material for: Comparative Transcriptome Analysis between Gynoecious and Monoecious Plants Identifies Regulatory Networks Controlling Sex Determination in Jatropha curcas
Source: Front Plant Sci. 2017 Jan 17;7:1953. doi: 10.3389/fpls.2016.01953 (PMC5239818; doi:10.3389/fpls.2016.01953)
Supplement: Supplementary file 9 [file Image_1.pdf]

Supplementary Figure S1

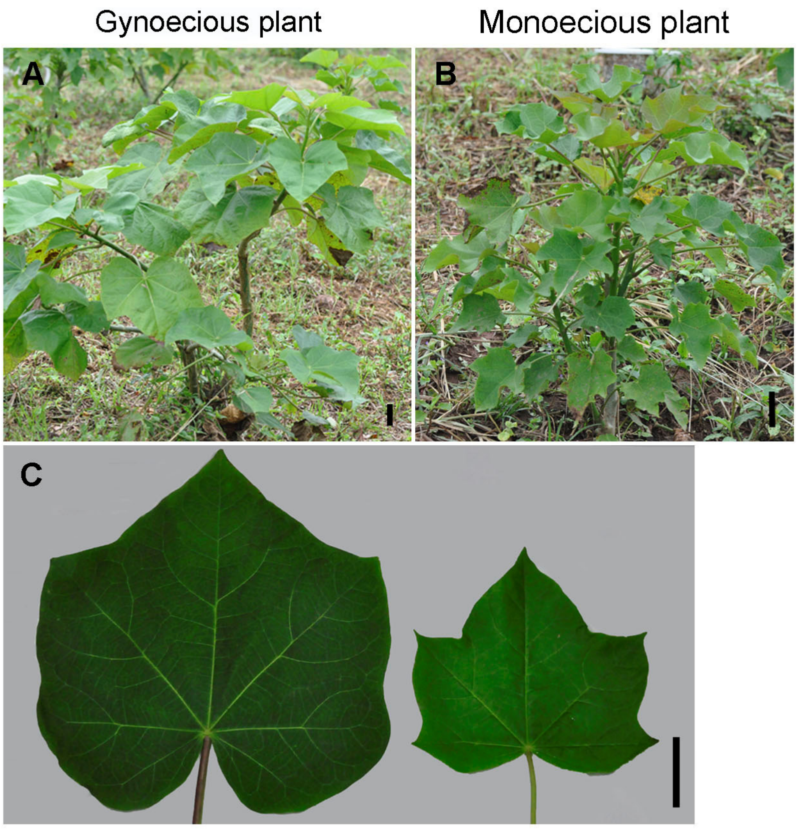

Figure S1 Phenotypes of gynoecious and monoecious *Jatropha* plants.
(A) Gynoecious plant; **(B)** Monoecious plant; **(C)** Leaf of gynoecious (left) and monoecious (right) *Jatropha*; bar = 5.0 cm.
