# Supplementary material for: Comparative Transcriptome Analysis between Gynoecious and Monoecious Plants Identifies Regulatory Networks Controlling Sex Determination in Jatropha curcas
Source: Front Plant Sci. 2017 Jan 17;7:1953. doi: 10.3389/fpls.2016.01953 (PMC5239818; doi:10.3389/fpls.2016.01953)
Supplement: Supplementary file 10 [file Image_2.pdf]

Supplementary Figure S2

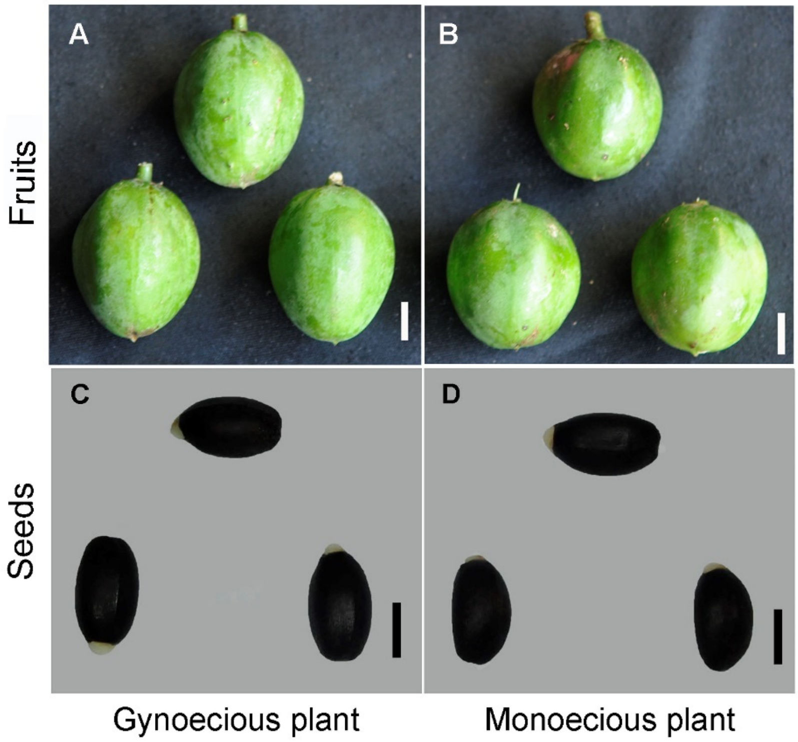

Figure S2 Comparison of fruits and seeds between gynoecious and monoecious *Jatropha*.

Fruits of gynoecious **(A)** and monoecious **(B)** plants**;** Seeds of gynoecious **(C)** and monoecious **(D)** plants; bar = 1.0 cm.
